# “I Dreamed of My Hands and Arms Moving Again”: A Case Series Investigating the Effect of Immersive Virtual Reality on Phantom Limb Pain Alleviation

**DOI:** 10.3389/fneur.2020.00876

**Published:** 2020-08-25

**Authors:** Xin Tong, Xinxing Wang, Yiyang Cai, Diane Gromala, Owen Williamson, Bifa Fan, Kunlin Wei

**Affiliations:** ^1^School of Interactive Arts and Technology, Simon Fraser University, Surrey, BC, Canada; ^2^China-Japan Friendship Hospital, Beijing, China; ^3^Department of Epidemiology and Preventive Medicine, Monash University, Melbourne, VIC, Australia; ^4^Motor Control Lab, School of Psychological and Cognitive Sciences, Peking University, Beijing, China

**Keywords:** immersive virtual reality, phantom limb pain, motor execution, motor imagery, brachial plexus nerve injury, serious games

## Abstract

Phantom limb pain (PLP) is a type of chronic pain that follows limb amputation, brachial plexus avulsion injury, or spinal cord injury. Treating PLP is a well-known challenge. Currently, virtual reality (VR) interventions are attracting increasing attention because they show promising analgesic effects. However, most previous studies of VR interventions were conducted with a limited number of patients in a single trial. Few studies explored questions such as how multiple VR sessions might affect pain over time, or if a patient's ability to move their phantom limb may affect their PLP. Here we recruited five PLP patients to practice two motor tasks for multiple VR sessions over 6 weeks. In VR, patients “inhabit” a virtual body or avatar, and the movements of their intact limbs are mirrored in the avatar, providing them with the illusion that their limbs respond as if they were both intact and functional. We found that repetitive exposure to our VR intervention led to reduced pain and improvements in anxiety, depression, and a sense of embodiment of the virtual body. Importantly, we also found that their ability to move their phantom limbs improved as quantified by shortened motor imagery time with the impaired limb. Although the limited sample size prevents us from performing a correlational analysis, our findings suggest that providing PLP patients with sensorimotor experience for the impaired limb in VR appears to offer long-term benefits for patients and that these benefits may be related to changes in their control of the phantom limbs' movement.

## Introduction

Phantom limb pain (PLP) is a type of chronic pain caused by limb amputation (1). Besides amputation, brachial plexus avulsion (BPA) injury—the detachment of the nerves from the nerve roots of the spinal cord in the arm—also leads to partial or complete arm paralysis and chronic pain (2). Most patients with BPA develop sensations in their damaged arm such as tingling, electric shock, and burning pain; this is similar to the PLP experienced by amputees (3). Therefore, researchers believe that studying BPA has the potential to deepen our understanding of the roles that the peripheral and central nervous systems play in PLP (4). The neural mechanism of PLP is still under debate. Some researchers proposed that cortical reorganization of neural representations of the missing limb and its neighboring body parts causes PLP (5–7). Others hold that the functional representation of the missing limb is preserved (8, 9), and “peripheral” contributors—such as neuroma formation and ectopic firing in the residual nerves—are the major contributors of PLP (10–12). It has also been proposed that impaired sensorimotor circuitry leads to PLP because both central and peripheral factors play a role (13, 14).

Researchers postulated that behavioral interventions for PLP might owe their analgesic effects to restoring the sensorimotor circuitry (15). These interventions usually provide augmented sensorimotor experience of the affected limb, including tactile stimulation (6) and surrogated visual representation (16). For example, in mirror therapies (MTs), the movements of the intact limb are reflected in a mirror, giving patients a vivid experience of their affected limb as if it is in motion (16). While critical reviews of MT find its analgesic effects are limited (17, 18), some researchers believe that this limitation is because the limb movements are restricted to the mirror surface (14). Combining virtual reality (VR) with MT has provided a better sense of embodiment of the phantom limb, including a sense of ownership (SoO) and a sense of agency (SoA) (19, 20) over their virtual body. In this article, the VR environment refers to immersive environments (21), where users are completely isolated from their physical surroundings and experience the three-dimensional virtual worlds through a stereographic head-mounted display (HMD). The resulting analgesic effects are comparatively stronger than those from traditional MT (22). However, most researchers focused only on the short-term analgesic effect from one VR session (20, 23). In fact, longitudinal studies on PLP used representations of a virtual limb displayed on a computer monitor instead of in immersive VR *per se* (24–26). Thus, longitudinal studies involving VR are still lacking.

With impaired sensorimotor circuitry, PLP patients also show degraded movement performance of the phantom limb. As a phantom limb is usually paralyzed or perceived as fixed in one or more particular positions (13), it is difficult for patients to imagine moving their phantom limbs visually. Thus, the capacity of motor imagery (e.g., the time a patient takes to perform a task) might serve as a measurement of movement performance of the phantom limb, given that similar activations in the motor cortex during motor imagery and actual movements were observed in healthy individuals (27). Indeed, previous studies demonstrated a prolonged response time and a lack of activation in the sensorimotor cortex during motor imagery tasks in amputees with PLP when compared to those without and that their response times, as well as activation, were closely related to the magnitude of the PLP (28, 29).

Here we examined the long-term effects of VR-based MT interventions on alleviating PLP and the accompanying changes in the motor imagery capacity involving the phantom limb. We hypothesized that the VR-MT interventions could simultaneously alleviate the pain and improve the motor imagery capacity for the phantom limb across multiple sessions.

## Materials and Methods

### Participants

We recruited five BPA and amputees' outpatients, all of whom were diagnosed with PLP (all male, age mean = 50.2, age SD = 7.73 years) from China-Japan Friendship Hospital in Beijing. All suffered from medium to severe levels of daily pain, and three of five have been taking the pain and/or antianxiety medicine. Detailed medical and demographic information is listed in Supplementary Table 1. For the inclusion criteria, we adopted similar standards as in a previous study (25): participants (1) need to be adults; (2) have been treated for PLP by at least one clinical approach; and (3) have not reported any pain changes for at least a year after the last session of prior treatments. Three patients exited the study before the planned 10 sessions because of their work and travel matters. They all signed the consent form and were informed that they could withdraw from the study without consequences. Each participant received monetary compensation. The Ethical Review Board of Peking University approved this study protocol (School of Psychological and Cognitive Sciences, #2018-06-02). Written informed consent was obtained from the participants for the publication of any potentially identifiable images or data included in this article.

### Setting and Apparatus

The immersive room-scale VR system and HMD were from HTC VIVE (30) with 1,080 × 1,200 pixels resolution per eye and a field of view of 110 degrees. Unity3D (31) software was used to develop the VR environment. Final IK Unity3D assets provide inverse kinematics' solutions for the avatar's body rigging and movement mapping (32). Participants saw the environment from a first-person perspective of a gender-matched avatar and remained seated during the entire study. The VR controller, held by the intact hand, and can register hand motion and button click.

### Instruments

We assessed the changes in pain ratings both before and after the VR intervention. Two pain ratings were used (1) Short-Form McGill Pain Questionnaire (SF-MPQ), which is the pain rating index (ratings from 0 to 75) formed by the summed contribution of 15 characteristics of pain (33); and (2) the visual analog scale (VAS) ratings from 0 to 10. Sense of embodiment (SoO and SoA) was rated once before the whole study and once after. Sense of ownership and SoA ratings were reported in an 11-point numerical rating scale (NRS) from 0 to 10, where 0 means “don't agree at all,” and 10 means “strongly agrees.” The SoO and SoA questions (Supplementary Table 2) were modified from related research (19). Further, the patients' depression and anxiety levels were measured using the Hospital Anxiety and Depression Scale (HADS) questionnaire (34) once before the entire study and once after.

### Procedures

**Figure 1:**
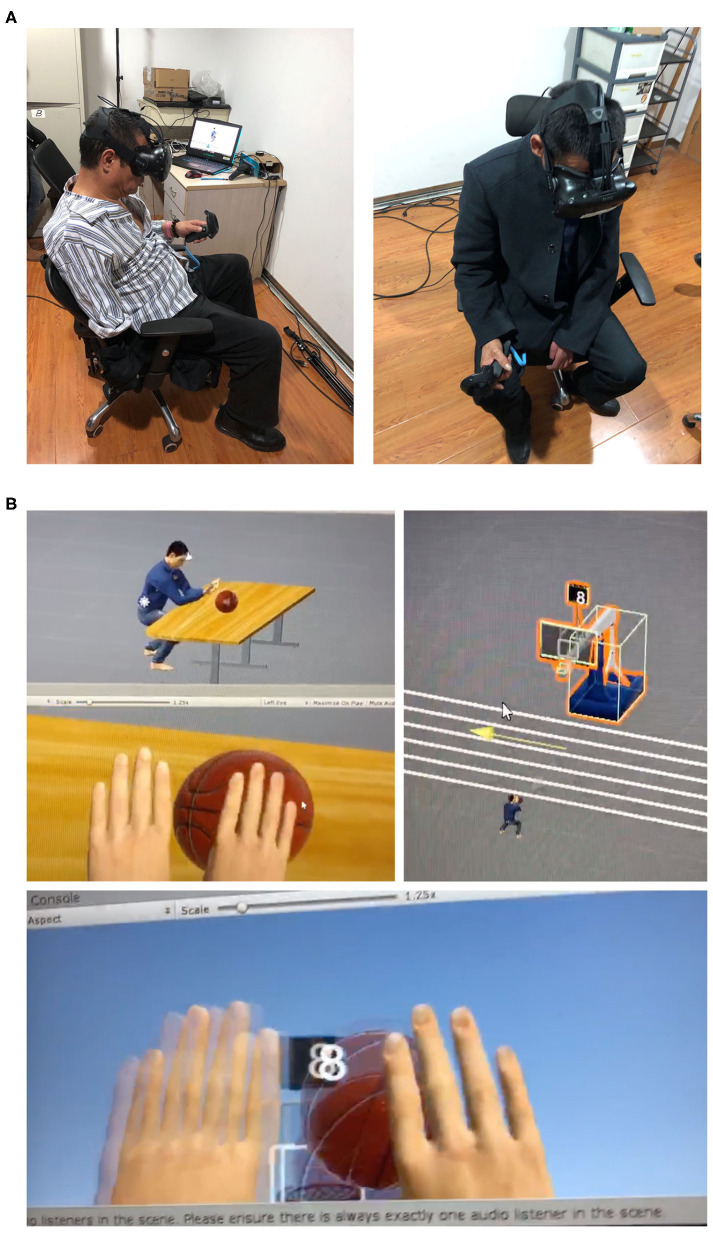
**(A)** Patients performing the ball-pushing task with an HTC VIVE's controller held in the intact hands (left: P04; right: P03). **(B)** The VR environment as depicted during the two tasks (the ball-pushing task and the ball-shooting task from third-person and first-person perspectives). Participants only saw the VR environment from the first-person perspective.

Each session lasted approximately 1 h with the following steps (Supplementary Figure 1):

1. The patient filled out the questionnaires for self-reported anxiety and depression ratings before session 1, and SoO and SoA ratings after session 1.
2. The researcher conducted semistructured interviews to collect the patients' subjective feedback before each session. The questions regarded (a) pain qualities and frequencies, (b) sleep quality, (c) medicine intake, (d) emotional changes, and (e) any other thoughts.
3. The patient filled out the two pretest pain questionnaires before each session.
4. The patient wore a VR HMD and held a controller in their intact hand, performing two motor tasks for 30 min (Figure 1).
5. The patient carried out the motor imagery and motor execution tasks, once before the first session and once after the last session. Before the former, the researchers detailed the task instructions before a practice session when patients performed the two VR motor tasks by execution and by imagery, three times each. The ball-pushing task required the participant to push a ball off the table with extension of both virtual limbs whose motion was driven by the measured motion of the intact limb only. The ball-shoot task is to extend both limbs to shoot a basketball toward a basket. Again, the motion of two limbs was driven by the intact limb only; the ball release was initiated by clicking the trigger button on the controller. The order of these practice runs (execution vs. imagery, ball-pushing vs. ball-shooting) was pseudorandomized across patients, and they performed each for three times per session. In the subsequent former test, patients were asked to visually imagine performing the two VR tasks with either limb (not both limbs); each task and each limb was repeated three times. They were instructed not to perform motor imagery unless they were told to. Patients then executed each task with the intact hand for three times. For each trial, the patient clicked the trigger button of the controller once before the trial, and once after the trial to register the time needed for imagery and execution.
6. The patient filled out the posttest VAS ratings after each session.
7. The patient filled out the questionnaires for self-reported anxiety and depression ratings, and SoO and SoA ratings immediately after the last session.

## Results

### Primary Outcomes—Pain Ratings

**Figure 2:**
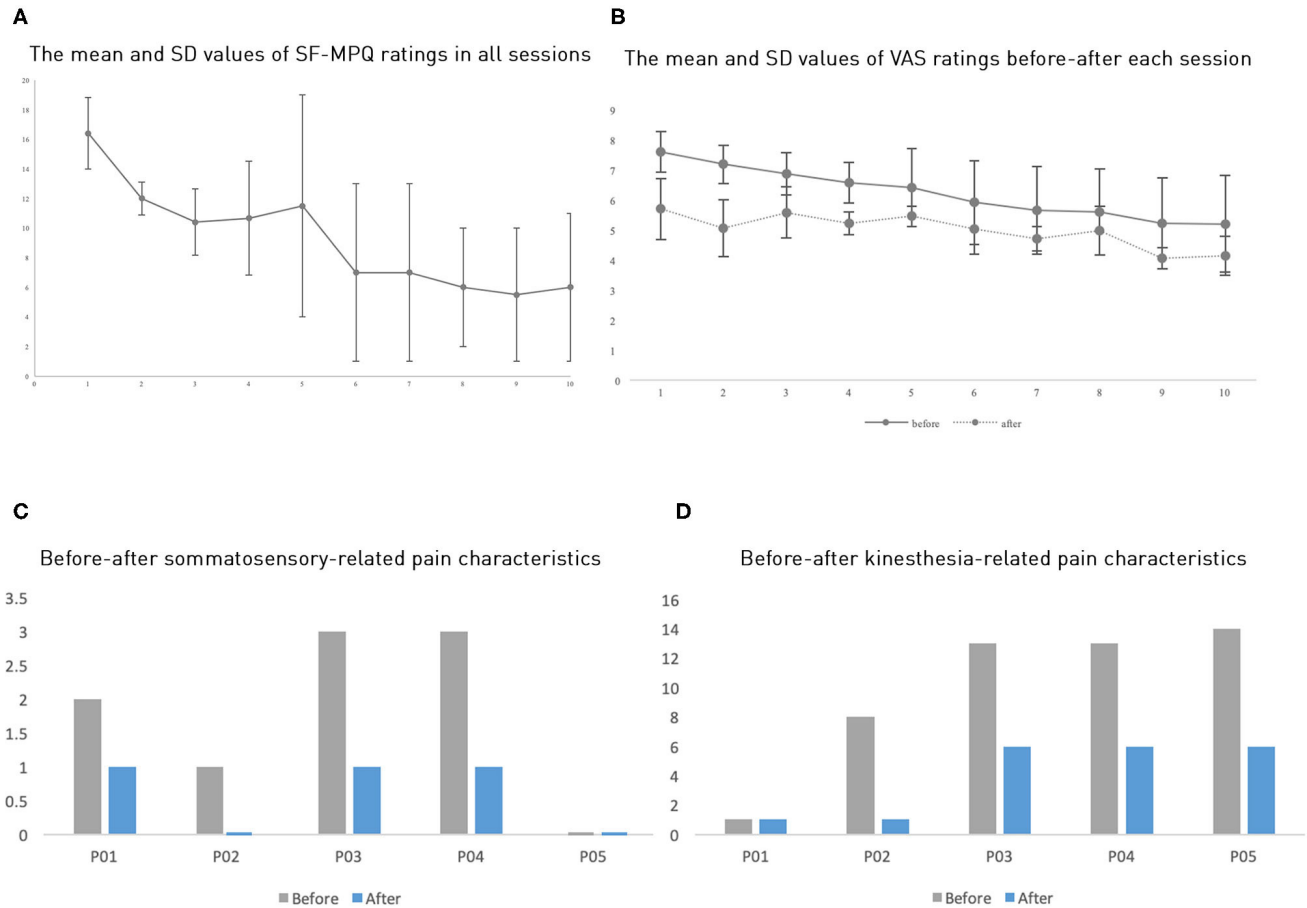
**(A)** The average SF-MPQ ratings across all sessions. **(B)** The average VAS ratings across sessions. **(C,D)** Each participant's ratings of somatosensory-related pain characteristics and kinesthesia-related characteristics (where P02 and P05 do not have bars meaning zero value). Here, error bars denote standard errors.

The pain ratings showed that all five patients had pain reduction, both before and after a session and across sessions (Table 1 and Figure 2). Patients P01 and P04 withdrew from the study after the third session, P5 after the fourth session; P2 and P3 completed all 10 sessions as planned. Because of the limited sample size, we opted to perform a non-parametric test to compare the pain ratings between the first session and the third session to examine whether the pain reduction was significant. The average of five patients' MPQ ratings was 16.4 (SD = 5.14) in the first session and 10.4 (SD = 5.03) in the third session, respectively. A Wilcoxon signed-rank test showed a significant improvement of pain rating in the third session compared to the first session with a large effect size despite the small sample size (*Z* = −2.02, *p* = 0.043, *r* = 0.9). Notably, all patients showed continuous pain reduction over consecutive sessions. Overall, patients reported an average improvement of 56.96% (SD = 17.49%) on the SF-MPQ ratings when comparing the last session, they took part in with their first session. Specifically, 56% improvement (SD = 18.08%) was on the pain sensation categories (throbbing, shooting, stabbing, sharp, cramping, gnawing, hot-burning, aching, heavy, tender, and splitting) and 58.33% (SD = 30.5%) on the emotional categories (tiring-exhausting, sickening, fearful, and cruel-punishing). Notably, all patients showed more than 50% improvement (ranging from about 50%, e.g., P01, to 90.91%, P02), although their initial pain ratings differed substantially (Figure 2B). Scrutinizing 15 pain qualities (Supplementary Figure 2), we found that all patients initially experienced and subsequently improved on emotional categories in their SF-MPQ ratings. For the sensory intensity category, four of the five patients shared throbbing, sharp, and heavy experiences; the heavy sensation disappeared after the intervention.

**Table 1:** Patients' pain reduction percentages between the first and last sessions of their participation of each individual and the group mean and standard deviation (SD) values.

	**No. of sessions participated**	**SF-MPQ rating reduction (%) (across sessions)**			**VAS (%)** **(across sessions)**	**VAS (%)** **(mean value before and after each session)**
		**Pain sensation** **categories**	**Emotional** **categories**	**Total**		
P01	3	42.86	66.67	49.21	25.36	20.6
P02	10	83.33	100	87.76	39.29	4.09
P03	10	38.46	66.67	45.98	9.89	28.79
P04	3	52.94	33.33	47.71	5.87	8.82
P05	4	64.71	25.00	54.12	14.79	43.86
Mean (SD)	6 (3.67)	56.48 (18.08)	58.33 (30.05)	56.96 (17.49)	19.04 (13.47)	21.23 (15.95)

Further, we also categorized the pain qualities into “kinesthesia-related pain characteristics” (splitting, exhausting, burning, aching, throbbing, stabbing, sharp, shooting) and “somatosensory-related pain characteristics” (gnawing, fearful, cramping), as a previous study found that VR mirror-movement therapy specifically improved the kinesthesia-related pain characteristics (20). However, we found that these two categories improved to a similar extent, with an average 50.47% (SD = 31.57%) and 56.67% (SD = 36.51%) improvement, respectively (Figures 2D,E).

The VAS ratings showed a similar but less drastic analgesic effect than the SF-MPQ ratings (Figure 2B and Table 1). The averages of the five patients' VAS ratings in the first three pretests were 7.6 (SD = 1.47), 7.19 (SD = 1.4), and 6.88 (SD = 1.56), whereas the posttests mean ratings were reduced to 5.71 (SD = 2.26), 5.07 (SD = 2.12), and 5.59 (SD = 1.91), respectively. The Wilcoxon signed-rank test showed that all three posttests had significantly reduced VAS ratings when compared to their corresponding pretests with a large effect size (for all three tests, *Z* = −2.02, *p* = 0.043, *r* = 0.9). Comparing VAS ratings across days, we found a marginally significant difference in pretest ratings between the first session and the third session (*Z* = −1.75, *p* = 0.08); however, the posttest ratings did not show a significant across-session difference (*Z* = −0.41, *p* = 0.68), possibly because the analgesic effect in each session masked the across-session differences. The average improvement of the VAS rating was 19.04% (SD = 13.47%). We found that each session induced an average improvement of 21.23% (SD = 15.95%) when comparing the pre-test VAS ratings with the posttest ones. All five participants showed this one-session improvement. Given the small sample size in this study, we would like to state the statistics should be viewed with caution.

### Phantom Limb Movement: Motor Imagery and Motor Execution Movement Time

**Figure 3:**
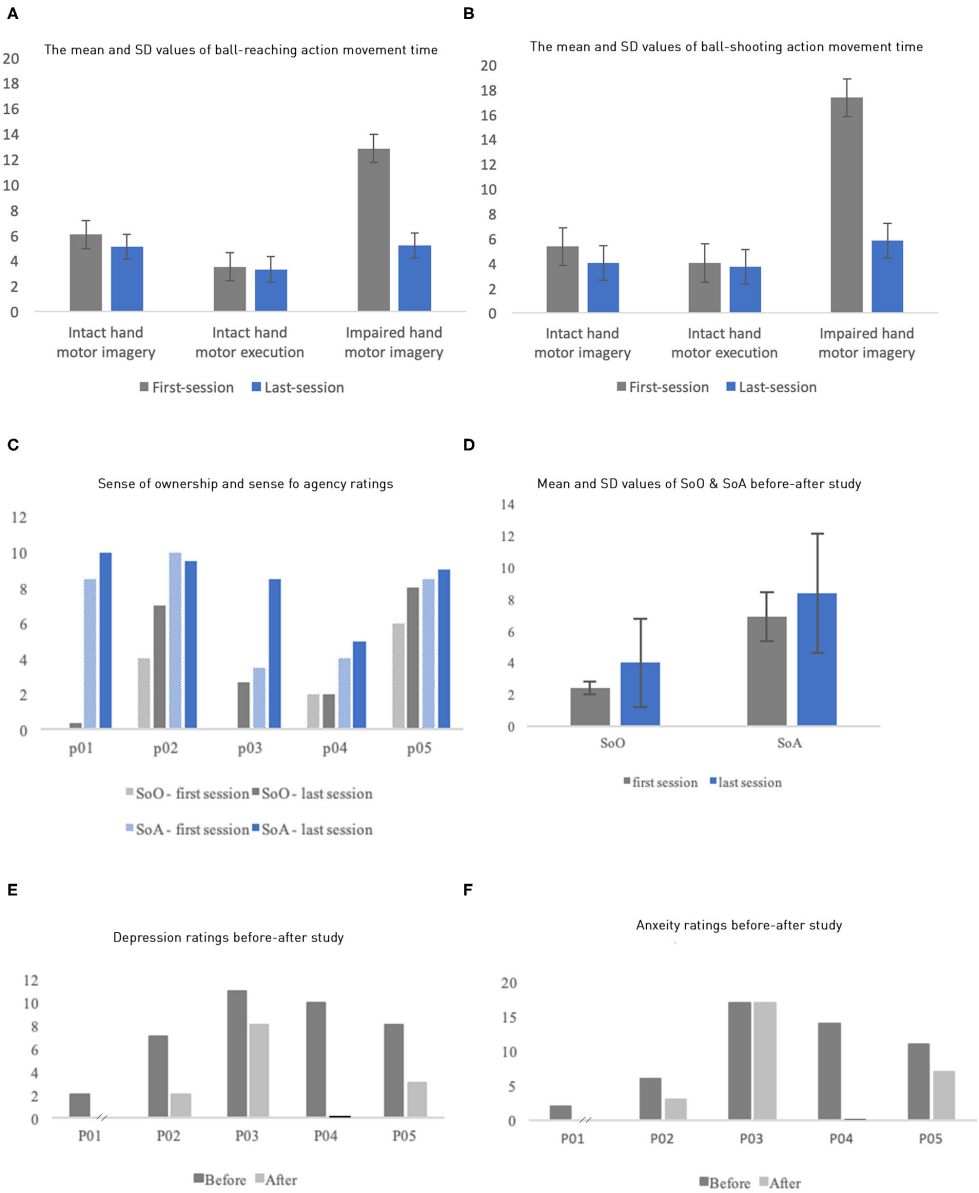
**(A,B)** Mean and SD of motor imagery time and motor execution time for the *ball-reaching* task by both the intact limbs and the impaired limbs (*y* axis: in seconds). **(F)** Similar results as **(E)** but for the ball-shooting task. **(C)** Each participant's sense of ownership and sense of agency ratings of their virtual body. **(D)** The mean SoO and SoA ratings of the first and the last sessions. **(E,F)** Patients' anxiety and depression ratings before and after the study (P01's after study depression and anxiety data were missing; P04's after-study rating means zero value).

The performance of motor imagery and execution was quantified by their movement time (Figures 3A,B; individual data in Supplementary Tables 3, 4). First, execution time and imagery time were similar for the intact limb, suggesting that participants followed our instruction. Both measures tended to decrease when measured again after the VR intervention, possibly due to a practice effect. As expected, we also observed that the impaired limb had substantially larger imagery time than the intact limb, with average of 12.83 ± 6.45 s and 17.23 ± 8.98 s for the ball-pushing and ball-shooting tasks, respectively. In contrast, the intact limb had average imagery time of 6.05 ± 3.30 s and 5.35 ± 1.79 s for these two tasks, respectively. Critically, the imagery time of the impaired limb was reduced dramatically after VR intervention, averaging 5.19 ± 3.84 s and 5.80 ± 4.48 s for the two tasks, respectively. These reductions, averages of 60.59 and 66.53%, brought the imagery time to the level comparable to that of the intact limb, suggesting that the phantom limb movement was dramatically improved after the intervention.

### Sense of Embodiment Ratings

The rating of SoO and SoA for the avatar in the VR increased in our experiment (Figures 3C,D). The ratings were measured twice through an 11-point NRS before and after all sessions, right after they took off the HMD. The questions for each category (Supplementary Table 2) were added up and averaged to one score per category. The SoO and SoA ratings increased, from the first to the last session, by 66.67 and 21.74%, respectively. Average SoA increased from 6.9 (first session, SD = 1.32) to 8.4 (last session, SD = 0.89); Correspondingly, average SoO increased from 2.4 (SD = 1.66) to 4.0 (SD = 1.48). However, P04's rating of SoO and P02's rating of SoA did not increase.

### Anxiety and Depression Ratings

The patients' anxiety and depression levels were measured using HADS, once before the first session, and once after the last session (Figures 3E,F). We missed the posttest ratings from P01 and P04 because they withdrew. All the remaining three patients experienced an improvement in anxiety and/or depression with varying degrees. P02 and P05 experienced an improvement in both the anxiety and the depression levels, whereas P03 showed improvement only on depression levels.

### Qualitative Interview Analysis

All patients reported one or a few positive changes after the intervention. Here, we report the qualitative results briefly. P01 said the VR intervention had provided him with an analgesic effect ranging from 2 h or longer until he went to bed at night. However, his anxiety from over 10 years of suffering hardly changed. P02 did not report a substantial change in pain before and after each intervention, but he did report a substantial decrease in pain ratings across the entire study. Furthermore, he reported multiple pain sensations in SF-MPQ initially, and only one at the study's conclusion. P03, before the study, reported over 30 times of “unbearable bursts of pain every day,” which he rated as 9 or 10 in VAS and lasted for 1 to 5 min. After the study, P03 reported that the intensity of his pain bursts was “much more endurable now” and that they lasted half the time. Notably, P03's quality of sleep steadily improved. Before participation, he woke up 8–10 times because of the pain bursts; at the conclusion of the study, he only woke up two to three times per night. P05's reported similar improvement in sleep: before the study, he reported, “I have problems falling asleep and I need to take pills. But now I don't need to.” Surprisingly, even though we did not ask, three out of five patients mentioned that they dreamt that their impaired limb moved again, the same way it had before their injury. According to P05, “I had a dream yesterday, and I saw my right hand and arm moving! It felt so good and so vivid that I can still remember.” Thus, these semistructured interviews showed that all five patients' subjective experiences are consistent with the quantitative measures, including pain ratings and motor imagery time.

## Discussion

Our brief report with five PLP patients reveals that a long-term VR-MT intervention produced substantial analgesia, indexed by SF-MQP and VAS pain ratings, along with improved phantom limb movement, quantified by reduced motor imagery time. Short-Form MQP and VAS ratings showed different percentages of improvement, given that they measure different aspects of pain perception with different levels of responsiveness (35, 36). We also found an enhanced sense of embodiment with the VR avatar and improved ratings in anxiety and depression. We observed all of these changes in each patient, although with varying effect sizes.

These findings suggest that VR-MT interventions hold promise as effective analgesia for patients who suffer PLP, particularly considering that four out of five participants suffered severe PLP for more than 10 years, and were first treated with at least one of the traditional pain management methods. Therefore, it is unlikely that carryover effects from previous therapies can explain our findings. For the same reason, pain relief owing to natural regression to the mean effects is unlikely to explain the observed large effect. Furthermore, patients who were taking medication had already been on it for over 2 years without an increase in dosage during the study; this makes medications an unlikely explanation for our results.

In our study, five patients underwent the VR intervention for 4–6 weeks, ranging from 3 to 10 sessions (Table 1). Previous VR studies mostly had a limited number of participants in longitudinal tests. For instance, Murray et al. (37) conducted a case study with three patients over two to five sessions; Henriksen et al. (38) investigated the feasibility of their VR environment with three upper limb amputees over seven sessions, and Chau and colleagues' case study involved only one PLP patient who participated in five sessions (39). Other VR studies involved a single session with one or more patients (20, 40–43). One reason that prevents large sample sizes is that patients with PLP usually need the help of caregivers to travel, and most patients lived far from the research laboratory (not in the same province). We also found that patients we initially tried to recruit were too physically inactive, mentally impaired, or socially disengaged to participate in the study.

While the potential of using VR for relieving PLP has been demonstrated, why and how it works remain unclear. Some researchers believe that having a sense of ownership over a virtual body in VR might alleviate pain for healthy subjects and pain patients (19, 44). Others proposed that VR distracts acute pain patients' attention from their pain by the multisensory, immersive VR environment (45–47). Both explanations received respective support. In fact, a combination of modified embodiment and distraction—by pairing a VR intervention with mindfulness meditation in order to direct attention inward to awareness of and agency over a patient's body—was shown as an effective intervention for chronic pain management (48). Our longitudinal data cannot be accounted for by distraction as the accumulated effect is obvious. We indeed observed more SoO and SoA, but their effect is relatively small.

With the growing evidence that the level of the phantom limb's movement may be correlated with a cortical or subcortical reorganization, others have also suggested that improved phantom limb movement may be associated with pain reduction (49). However, in only one study was the phantom limb's movement actually measured quantitatively (20). Our data here also showed an improvement in movements of a phantom limb, quantified as a reduction in motor imagery time that was specific to the impaired limb. Given that the motor imagery was measured only twice, we believe that the practice effect alone could not explain the large and limb-specific effect. The observed 60.59 and 66.53% reduction in imagery time in the two motor tasks was remarkable because it dropped to levels comparable to that of the intact limb. The improvement suggests better control of the impaired limbs' movement. Osumi and colleagues used a bimanual coupling effect between the affected limb and the intact limb as an indirect measure of changes in phantom limb control. They found that bimanual coupling increased with VR interventions and, importantly, were correlated with the VR-induced analgesic effect. Our findings of improved motor imagery in the affected limb are in line with Osumi et al. (20) findings, suggesting that improved voluntary movement of the phantom limb might reflect the neuroplastic changes in PLP patients that are associated with VR's analgesic effects. However, we did not run a correlation analysis between the improvement in motor imagery and the analgesic effect due to the small sample size.

The first limitation of this study is the small sample size which prevents us from establishing the correlation between pain reduction and accompanied changes in the phantom limb movement and embodiment. In future studies, we plan to conduct a longitudinal controlled trial with more samples and methodological improvements. For example, a motor imagery test can be performed measuring electromyography in residual muscles. Sense of agency and SoO can be potentially quantified by more objective approaches, such as intentional binding. We could also compare VR interventions without or without a virtual body. The VR experience can be complemented with haptic feedback to enhance embodiment (50). Importantly, the improvement in the phantom limb movement, as revealed by motor imagery time, can be further investigated by electroencephalogram or functional magnetic resonance imaging scans to probe possible neural reorganization brought about by VR interventions.

## Data Availability Statement

All datasets generated for this study are included in the article/Supplementary Material.

## Ethics Statement

The studies involving human participants were reviewed and approved by the Ethical Review Board of Peking University. The patients/participants provided their written informed consent to participate in this study.

## Consent for Publication

Written informed consent was obtained from the participants for the publication of any potentially identifiable images or data included in this article.

## Author Contributions

XT and KW designed the study and the VR environment. XT and XW conducted this research study. XT, KW, YC, DG, XW, and BF wrote and revised the paper. XW and BF recruited participants. OW gave suggestions to the VR environment design and revised the paper. All authors contributed to the article and approved the submitted version.

## Conflict of Interest

The authors declare that the research was conducted in the absence of any commercial or financial relationships that could be construed as a potential conflict of interest.
