# Supplementary material for: “I Dreamed of My Hands and Arms Moving Again”: A Case Series Investigating the Effect of Immersive Virtual Reality on Phantom Limb Pain Alleviation
Source: Front Neurol. 2020 Aug 25;11:876. doi: 10.3389/fneur.2020.00876 (PMC7477390; doi:10.3389/fneur.2020.00876)
Supplement: Supplementary file 1 [file Data_Sheet_1.docx]

Supplementary Material

# Supplementary Tables

|  | Gender & Age  (years old) | Injury Cause | Injury | Injury Time (years) | PLP Time  (years) | VAS score pre-treatment  Out of 10 | Previous Treatment | Current  Medication | Time on Medication  (years) |
| --- | --- | --- | --- | --- | --- | --- | --- | --- | --- |
| Patient 1 (P01) | Male  37 | Car Accident | BPA (Right Arm) | 1 | 1 | 6.86 | PRDRG | Gabapentin | 1 |
| Patient 2 (P02) | Male  50 | Car Accident | BPA (Right  Arm) | 18 | 18 | 5.93 | DREZL | N/A (because medicine won’t help) | N/A |
| Patient 3 (P03) | Male  56 | Car Accident | BPA (Left  Arm) | 19 | 19 | 7.44 | PRDRG | Gabapentin,Estazolam Flupentixol & Melitracen Tablets | 5 |
| Patient 4  (P04) | Male  55 | Car Accident | Amputation  (Right Arm ,10cm left left) | 10 | 10 | 9.88 | NRB | Gabapentin | 10 |
| Patient 5  (P05) | Male  53 | Car Accident | BPA (Left  Arm) | 30 | 30 | 7.91 | NRB | No pain medicine (because it won’t work) but melatonin (for sleep problem) | N/A |

**Supplementary Table 1.** Patients’ Demographics and medical information (PRDRG = Pulsed Radiofrequency on Dorsal Root Ganglion; DREZL = dorsal root entry zone lesioning; NRB = Nerve Root Block)

| Category | Questions: During the current experiment condition in Virtual Reality, |
| --- | --- |
| Body Ownership | 1. I feel as if the virtual arms and hands were my own real arms and hands.  2. I feel my real arms and hands were becoming virtual.  3. I feel my real arms and hands were moving some times. |
| Body Agency | 4. I feel as if the virtual hands have a will on their own.  5. I feel the virtual fingers and hands would move in the same way as my real fingers and hands. |

**Table 2.** The SoO and the SoA questionnaire sorted by categories.

|  | Mean and SD of the Reaching Action Reaction Time (s) | | | | | |
| --- | --- | --- | --- | --- | --- | --- |
|  | The First Session | | | The Last Session **(% of changes compared to the first session)** | | |
|  | Motor Imagery  the impaired hand | Motor Imagery the intact hand | Motor Execution  the intact hand | Motor Imagery  the impaired hand | Motor Imagery  the intact hand | Motor Execution  the intact hand |
| P02 | 3.929 | 1.21 | 1.51 | 2.457 (37.47) | 2.041(-68.68) | 1.55 (-2.65) |
| P03 | 17.376 | 7.273 | 1.92 | 3.402 (80.42) | 2.619 (63.99) | 1.99 (-3.65) |
| P04 | 17.864 | 7.11 | 4.488 | 10.867 (39.17) | 6.58 (7.45) | 3.929 (12.46) |
| P05 | 17.791 | 10.624 | 9.178 | 3.006 (83.1) | 3.154 ( 12.46) | 3.154 ( 65.64) |
| **Mean** | **14.24** | **6.554** | **4.274** | **4.933 (60.04)** | **3.599 (18.3)** | **2.656 (17.95)** |
| **SD** | **6.877** | **3.914** | **3.525** | **3.975 (25.12)** | **2.039 (64.49)** | **1.086 (32.63)** |

**Table 3.** Patients’ mean and SD values of their impaired and intact hands’ motor imagery reaction time and the intact hand’s motor execution reaction time in millisecond at the first and last sessions of their participation of each individual and the group mean (reaching action).

|  | Mean and SD of the Shooting Action Reaction Time (s) | | | | | |
| --- | --- | --- | --- | --- | --- | --- |
|  | The First Session | | | The Last Session **(% of changes compared to the first session)** | | |
|  | Motor Imagery  the impaired hand | Motor Imagery  the intact hand | Motor Execution  the intact hand | Motor Imagery  the impaired hand | Motor Imagery  the intact hand | Motor Execution  the intact hand |
| P02 | 4.988 | 4.081 | 0.9 | 2.573 (48.42) | 1.842 (54.86) | 0.94 ( -4.44) |
| P03 | 29.781 | 7.136 | 1.88 | 3.383 (88.64) | 2.444 (65.75) | 1.67 ( 11.17) |
| P04 | 18.28 | 7.387 | 6.047 | 12.357 (32.4) | 7.287 (1.35) | 6.242 (3.22) |
| P05 | 23.574 | 3.573 | 3.55 | 4.901 (79.21) | 4.454 (24.66) | 4.565 (28.59) |
| **Mean** | **19.156** | **5.544** | **3.094** | **5.804 (62.17)** | **4.007 (24.33)** | **3.354 (6.27)** |
| **SD** | **10.55** | **1.996** | **2.252** | **4.474 (26.24)** | **2.455 (43.11)** | **2.481(16.48)** |

**Table 4.** Patients’ mean and SD values of their impaired and intact hands’ motor imagery reaction time (in seconds) and the intact hand’s motor execution reaction time in millisecond at the first and last sessions of their participation of each individual and the group mean (shooting action).

# Supplementary Figures


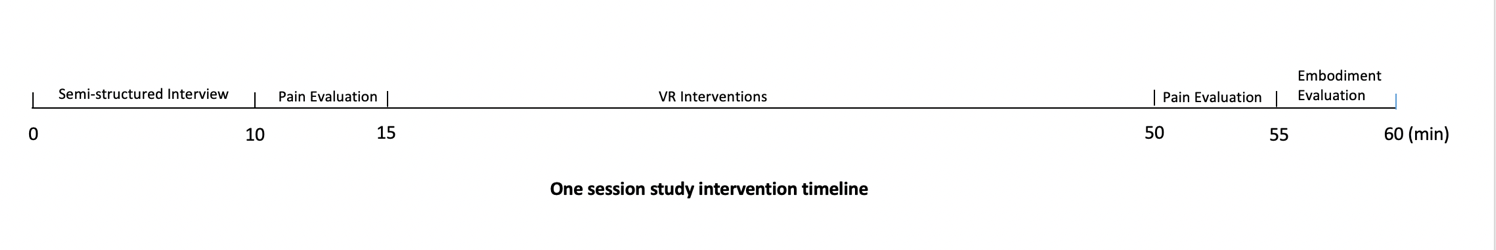


**Supplementary Figure 1.** Study procedure timeline for every single session.


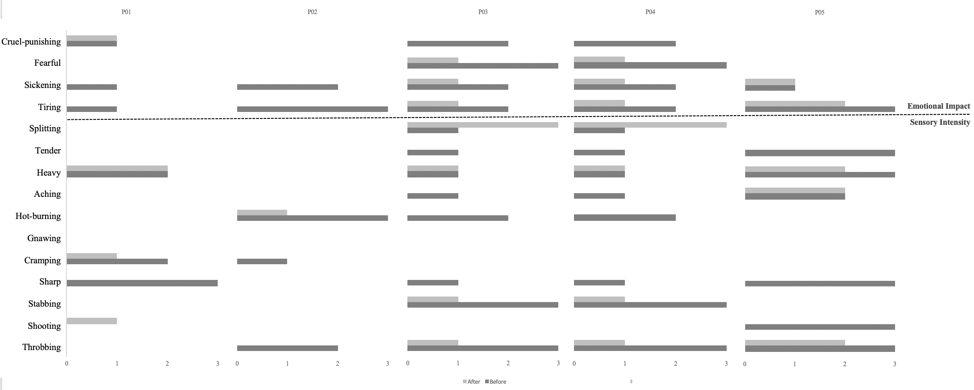


**Supplementary Figure 2.** Each participants’ SF-MPQ data, containing 15 pain qualities
